# SASHAYDIALL: A SAS Program for Hayman’s Diallel Analysis

**DOI:** 10.2135/cropsci2018.01.0047

**Published:** 2018-06-14

**Authors:** Dan Makumbi, Gregorio Alvarado, José Crossa, Juan Burgueño

**Affiliations:** 1 International Maize and Wheat Improvement Center (CIMMYT), PO Box 1041-00621, Nairobi, Kenya; 2 Biometrics and Statistics Unit, International Maize and Wheat Improvement Center (CIMMYT), Apdo. Postal 6-641, 06600, Mexico DF, Mexico

## Abstract

Different methods of diallel crossing are commonly used in plant breeding. The diallel cross analysis method proposed by Hayman is particularly useful because it provides information, among others, on additive and dominance effects of genes, average degree of dominance, proportion of dominance, direction of dominance, distribution of genes, maternal and reciprocal effects, number of groups of genes that control a trait and exhibit dominance, ratio of dominant to recessive alleles in all the parents, and broad-sense and narrow-sense heritability. In this paper, we fully describe a SAS-based software SASHAYDIALL for performing a complete diallel cross analysis based on Hayman’s model with or without reciprocals. We demonstrate the use of SASHAYDIALL with two data sets; one is a published diallel cross data set with reciprocals in cabbage (*Brassica oleracea* L.), and the second is a data set from a multilocation diallel cross trial in maize (*Zea mays* L.) without reciprocals. With SASHAYDIALL, diallel experiments conducted in single sites can be analyzed to estimate various genetic parameters, and this analysis is extended over locations or environments to assess genetic effect × environment interaction. SASHAYDIALL is user-friendly software that provides detailed genetic information from diallel crosses involving any number of parents and locations.

**THE DIALLEL CROSS**, made by crossing a set of genotypes in all possible combinations, is one of the most popular mating designs used in plant breeding. There are variations of the diallel depending on whether the parents and reciprocals are evaluated together with the F_1_s. Different methods of diallel cross analysis were developed by Jinks and Hayman (1953), Hayman (1954a, 1954b), Griffing (1956), and Gardner and Eberhart (1966). The most commonly used method of diallel analysis is the one developed by Griffing (1956), in which four different methods of analyses were proposed. Griffing’s (1956) methods of analysis provide estimates of general (GCA) and specific combining ability (SCA), variance components, and information on maternal and reciprocal effects. The Gardner and Eberhart (1966) method of analysis is applied in generation mean analyses of populations and provides information on additive and nonadditive genetic effects, heterosis, and inbreeding depression. The analyses proposed by Jinks and Hayman (1953) and described in more detail by Hayman (1954a, 1954b) are robust in terms of genetic information generated and provide information on additive and dominance effects of genes, average degree of dominance, proportion of dominance, direction of dominance, distribution of genes, maternal and reciprocal effects, number of groups of genes that control a trait and exhibit dominance, ratio of dominant to recessive alleles in all the parents, and broad-sense (*H*^2^) and narrow-sense (*h*^2^) heritability.

Hayman’s (1954a, 1954b) diallel analysis method has been used to study the mode of inheritance of various traits in several crops including barley (*Hordeum vulgare* L.) (Johnson, 1963; Riggs and Hayter, 1972, 1973; Jana, 1975; Olsen, 1979; Douglas and Gordon, 1985; Caligari et al., 1987; Komatsuda et al., 1989), *Brassicas* (Ono and Takahata, 2000; Zhang and Takahata, 2001; Sparrow et al., 2004a, 2004b; Tanaka and Niikura, 2006; Horisaki and Niikura, 2007), cotton (*Gossypium hirsutum* L.) (Verhalen et al., 1971; Innes et al., 1974; Luckett, 1989; Zhang et al., 2007), maize (Zea mays L.) (Rood and Major, 1981a, 1981b; Hohls et al., 1996; Betrán et al., 2003; Srdić et al., 2007; Kagoda et al., 2011), pea (*Pisum sativum* L.) (Bencheikh and Gallais, 1996), peanut (*Arachis hypogaea* L.) (Gibori et al., 1978), radish (*Raphanus sativus* L.) (Iwata et al., 2000, 2004), rice (Oryza sativa L.) (Mohanty and Khush, 1985; Xu and Shen, 1991; Pooni et al., 1993; Chaubey et al., 1994; Kuo et al., 1997; Taguchi-Shiobara et al., 1997; Nemoto et al., 2004), soybean [*Glycine max* (L.) Merr.] (Spehar, 1995; Spehar and Galwey, 1996; Tukamuhabwa et al., 2002), sugarbeet (Beta vulgaris L.) (Jolliffe and Arthur, 1993), tobacco (*Nicotiana rustica* L.) (Jinks, 1954; Pooni et al., 1997), triticale (× *Triticosecale* Wittmack) (Mather and Poysa, 1983; Carrillo et al., 1985), and wheat (*Triticum aestivum* L.) (Crumpacker and Allard, 1962; Klaimi and Qualset, 1973; Law et al., 1978; Danon and Eyal, 1990; Ghannadha et al., 1995; Wagoire et al., 1998), among others.

Specialized software has been developed for Griffing’s diallel analysis (Shattuck et al., 1993; Burow and Coors, 1994; Magari and Kang, 1994; Zhang and Kang, 1997; Wu and Matheson, 2000; Zhang et al., 2005; Möhring et al., 2011) and Gardener–Eberhart analysis (Murray et al., 2003; Zhang et al., 2005), but very few packages other than those by Lee and Kaltsikes (1971), Ukai (1989), and GenStat (VSN International, 2015) have been specially developed for Hayman’s diallel analysis. The lack of easy-to-use specialized software for the Hayman (1954b) diallel analysis method limits its application in plant breeding. Currently,we have no information on a widely available program in SAS (SAS Institute, 2014) to analyze a diallel cross data set from multiple locations or environments according to Hayman’s method with comprehensive output. Therefore, there is a need to fill the gap through development of an SAS-based software program to analyze data according to the method proposed by Hayman (1954b). In this paper, we (i) describe a SAS program SASHAYDIALL developed for analysis of diallel cross data according to Hayman’s (1954b) methodology, and (ii) demonstrate the use of SASHAYDIALL with two diallel cross data sets.

## Theory of Hayman’s Diallel Analysis

Hayman’s (1954b) method of diallel analysis involves the estimation of genetic parameters, and graphical and statistical analyses of array variances and covariances. The key assumptions for analysis of diallel cross data based on Hayman’s method are (i) diploid segregation, (ii) homozygous parents, (iii) no differences between reciprocal crosses, (iv) no epistasis, (v) no multiple alleles, and (vi) independent distribution of genes among the parents (Jinks and Hayman, 1953; Hayman, 1954a, 1954b).

Considering that an “array” refers to all the crosses involving a particular parent, the steps required to carry out Hayman’s diallel analysis are (i) ANOVA to detect genetic variation among the genotypes, (ii) creation of the array of means in the diallel table, (iii) calculation of the expected variance (*V*_ri_) and parent–offspring covariance (*W*_ri_) of individual arrays, (iv) calculation of the mean variance ($\left({\bar{V}}_{r}\right)$) and covariance ($\left({\bar{W}}_{r}\right)$) over all arrays, v) calculation of the variance of the array means ($\left({\bar{V}}_{r}\right)$), (vi) testing the validity of the additive–dominance model, (vii) generation of the *W*_r_ − *V*_r_ graph, and (viii) estimation of the genetic components.

In Hayman’s method of diallel cross analysis, six variances and covariances are calculated from the data set. These are the variance among parents (*V*_P_); the variance among family (*V*_r_) (F_1_ + reciprocal) means within an array; the mean value of *V*_r_ over all arrays ($\left({\bar{V}}_{r}\right)$); the variance among the means of the arrays ($\left(V_{\bar{r}}\right)$); the covariance between families within the *i*th array and their nonrecurrent parent (*W*_r_), and the mean value of *W*_r_ over all arrays ($\left({\bar{W}}_{r}\right)$). According to the theory of Hayman, the parameters for additive (*D*), and dominance gene effects (*H*), and the distribution of genes (*F*) are defined as:

*D* = 4Σ*uvd*^2^

*H*_1_ = 4Σ*uvh*^2^

*H*_2_ = 16Σ*u*^2^*v*^2^*h*^2^

*F* = 8Σ*uv*(*u* − *v*)*dh*

in which *u* and *v* are the frequencies of increasing and decreasing alleles, respectively, and *u* + *v* = 1, *d* represents the gene’s contribution to the fixable or additive genetic variation, and *h* is the difference between the heterozygote and the mid-homozygote values (Hayman, 1954a, 1954b). In Hayman’s diallel analysis, four second-degree statistics are calculated from the parents and F_1_ progeny. These are *V*_0*L*0_ (variance of parents), *V*_1*L*1_ (mean variance of arrays), *V*_0*L*1_ (variance of the array means), and *W*_0*L*01_ (mean covariance of array means) (Hayman, 1954a, 1954b). These seconddegree statistics are related to the three genetic components of the variation as shown below (Hayman, 1954b):

*V*_0*L*0_ = *D*

*V*_1*L*1_ = 1/4*D* + 1/4*H*_1_ − 1/4*F*

*V*_0_*_L_*_1_ = 1/4*D* + 1/4*H*_1_ − 1/4*H*_2_ − 1/4*F*

*W*_0*L*01_ = 1/2*D* − 1/4F

The ANOVA of a diallel table according to Hayman includes main effects denoted *a* (additive genetic effects), *b* (dominance genetic effects), *c* (average maternal effects of each parental line), and *d* (variation in the reciprocal differences not attributed to *c*) (Hayman, 1954a). The main effect *b* is further partitioned into three effects, namely, *b*_1_ (test of mean deviation of F_1_ from their mid-parental values), *b*_2_ (test of whether mean dominance deviation of the F_1_ from their mid-parental values within each array differs over arrays), and *b*_3_ (test of dominance deviation that is unique to each F_1_) (Hayman, 1954b; Mather and Jinks, 1971). The analysis developed by Hayman is related to that of Griffing (1956), but the two methods differ in the genetic assumptions and interpretations. Griffing’s GCA, SCA, and reciprocal effects components are equivalent to Hayman’s *a*, *b*, and (*c* + *d*) components, respectively (Mather and Poysa, 1983). The analysis proposed by Hayman required a complete diallel, but Morley Jones (1965) extended Hayman’s analysis for the half-diallel. Hayman’s method of diallel analysis also includes graphical analysis, whereby *W*_r_ is plotted against *V*_r_. In the *W*_r_ − *V*_r_ graph, the dominance order of the parents can be inferred from the relative position of the array points along the regression line of *W*_r_ on *V*_r_. The intercept of the regression line on *W*_r_ provides information on the degree of dominance in the genetic material under question.

## PROGRAM DESCRIPTION

The SASHAYDIALL program was written in SAS/IML (SAS Institute, 2013) and runs in SAS (SAS Institute, 2014). SASHAYDIALL consists of codes that correspond to the steps necessary to execute diallel cross analysis according to Hayman (1954a, 1954b). The linear model for Hayman’s diallel analysis implemented in SASHAYDIALL program is shown in the equations below:

$$\gamma_{rs}=m+j_{r}+l+l_{r}+l_{s}+l_{rs}+k_{r}-k_{s}+k_{rs}(r\neq s)$$ [1]

$$\gamma_{r}=m+2j_{r}-(n-1)l-(n-2)l_{r}(r=s)$$ [2]

where *γ_rs_* is the entry in the *r*th row (female parents) and *s*th column (male parents), *m* is the grand mean of the diallel table, *j_r_* is mean deviation from the grand mean due to the *r*th parents, *l* is mean dominance deviation, *l_r_* is further dominance deviation due to the *r*th parent, *l_rs_* is the remaining discrepancy in the *rs*th reciprocal sum, *k_r_* is average maternal effect of each parental line, and *k_rs_* is the variation in the *rs*th reciprocal differences (Hayman, 1954a). The parameters in the model measure different sources of variation whereby *j_r_* = *a* (variation due to additive genes), *l* = *b*_1_ (mean dominance deviation), *l_r_* = *b*_2_ (further dominance deviation due to the *r*th parental line), *l_rs_* = *b*_3_ (residual dominance variation), *k_r_* = *c* (average maternal effects of each parental line), and *k_rs_* = *d* (variation in the reciprocal differences not due to *c*) (Hayman, 1954a).

**Fig. 1.**
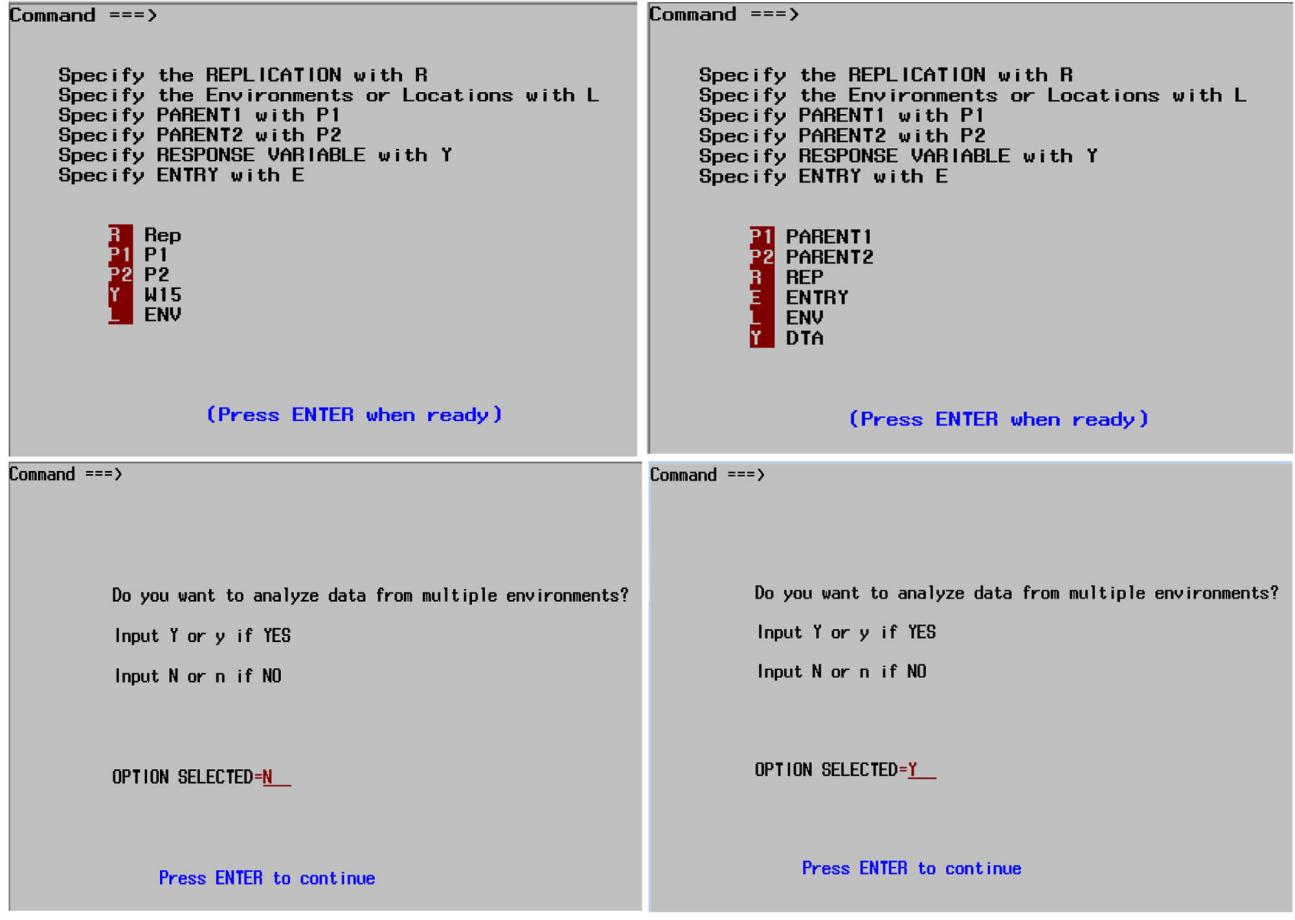
Input windows for SASHAYDIALL with different data arrangements. SASHAYDIALL will read the variables in the data set, but the user is required to give the corresponding codes, specify the response variable to be analyzed, and indicate whether to analyze single or multiple environment data.

The SASHAYDIALL program is based on the mathematical derivations presented in Hayman (1954a, 1954b) and Mather and Jinks (1971). For the analysis to proceed, the SASHAYDIALL program requires an input “csv” format data file that has replication (REP), Parent 1, Parent 2, and the trait of interest for a single site experiment. The data are arranged as REP P1 P2 VAR where VAR is the name of the trait to be analyzed. For data obtained from multiple environments, the user should include a variable for the locations, and the arrangement could be REP P1 P2 ENV VAR, but any order can be provided by the user. The user can include another variable “ENTRY” if they so wish, but this is not required by SASHAYDIALL for analysis. It is important that locations or environments are numbered sequentially in the “csv” file. The file with data to be analyzed can be saved in any directory. The user is only required to specify the location and name of the data file to be analyzed in the SASHAYDIALL program. The program automatically detects the number of parents and presence or absence of reciprocals in the diallel cross data set. Before analysis can proceed, the user is required to provide responses in two input windows: one window for variable information, and the other to indicate whether the data to be analyzed are from single or multiple environments (Fig. 1).

As a first step, a general ANOVA of the data using both PROC MIXED and PROC GLM (for single and across locations or environments) is executed to detect differences among genotypes. The SASHAYDIALL program will then ran ANOVA for the diallel table in presence or absence of reciprocals. The main effects in the ANOVA are tested for significance using both their interaction with replication and the residual as the error terms, and output for both cases is provided. The main effects can be tested against the residual if the error variances are homogenous (Mather and Jinks, 1971). The SASHAYDIALL program will then execute various computations as described by Hayman (1954b), including creation of an array of variances (*V*_ri_) and covariances (*W*_ri_), calculation of the four second-degree statistics, and testing the adequacy of the additive–dominance model using a *t* test. Estimates of genetic parameters including *D*, *H*_1_, *H*_2_, *F*, *H*^2^, *h*^2^, and the average degree of dominance, among others, are computed by SASHAYDIALL. The heritability estimates (*H*^2^ and *h*^2^) are computed using the formulae given by Mather and Jinks (1971). Finally, regression analysis for *W*r on *V*r is computed and a *W*_r_ − *V*_r_ graph is generated by SASHAYDIALL. A *W*_r_ + *V*_r_ vs. *Y*_r_ (the mean parental value) graph is also plotted. These computations are performed for a single site and across locations or environments, depending on the data set. The SASHAYDIALL program is not computationally intensive.

Below, we provide limited parts of the SASHAYDIALL program code to show different steps followed in Hayman’s (1954b) method of diallel analysis. Brief comments are provided to guide readers on the functions of some of the SAS statements in the code. We also provide part of a maize diallel data set from multiple environments to show data arrangement for analysis using SASHAYDIALL (see Supplemental Table S1).

**%let dir = D:\** /*specify location of file with data to be analyzed*/

;

**%let FileName** = MAIZEDIALLEL /*name of data file to be analyzed*/

;

PROC IMPORT datafile=”&dir\&FileName..csv” /*to import the data file*/

out=DIALLEL

dbms=csv

replace;

getnames=yes;

**RUN**;

**Data** DIALLEL;

set DIALLEL;

dsid=open(‘DIALLEL’);

if varnum(dsid, ‘Env’)=**0** then Env=**1**;

rc=close(dsid);

drop dsid rc;

**RUN**;

%global Y Parent1 Parent1 replication genotype Env;

**%macro** testVAR(var);

%let dsidvar=%sysfunc(open(&var));

%let nvars=%sysfunc(attrn(&dsidvar,nvars));

**%mend** testVAR;

**%mend** listsVAR;

**Data** DIALLEL;

set DIALLEL (rename=(&varsvar));

Genotype=compress(Parent1||”x”||Parent2); /*make a list of genotypes*/

**RUN**;

**PROC SORT** data=DIALLEL; /*sort data in the file*/ by Env Parent1 Parent2 Replication;

**PROC MEANS** data = DIALLEL mean noprint;

/*calculate trait means*/

by Parent1 Parent2;

var Response;

output out = tmp1 (drop = _type_ _freq_) mean= Response;

*----------------------------------------------------*

*General ANOVA for the response variable*

*----------------------------------------------------*

*The statements below are for execution of ANOVA for the trait of interest as specified by the user*

*---------------------------------------------------*;

**PROC MIXED** data=DIALLEL method=type3 noinfo noitprint;

class Env REP Parent1 Parent2 Genotype;

Model Response = Env Genotype Genotype*Env;

random REP(Env);

ods output type3=allc;

ods output ClassLevels=niv /*(keep = class level values)*/;

ods listing exclude FitStatistics ClassLevels type3 tests3 covparms;

**RUN**;

*---------------------------------------------------*

*ANOVA according to Hayman’s method*

*----------------------------------------------------*

*The statements below are for execution of ANOVA for the trait of interest among the F_1_s according to Hayman (1954b)*

*---------------------------------------------------*;

**PROC GLM** data=diallel outstat=temp2 noprint;

class Env replication Parent1 Parent2;

Model Response = Replication(Env) Replication Parent1 Parent2 Parent1*Parent2 Env Env*Parent1 Env*Parent1 Env*Parent1*Parent2/ss3;

**RUN**;

*----------------------------------------------------*

*Creation of data set for each parent*

*----------------------------------------------------*

*The next set of macros are used to create a data set for each parent in the diallel*

*---------------------------------------------------*;

%**macro *matrix***;

%do i=**1** %to &P;

data C&I;

set MY;

if P1=&i OR P2=&i;

rename MY=C&i;

drop P1 P2;

%if &i=2 %then %do;

data all;

merge C1 C2;

%end;

%if &I>2 %then %do;

data all;

merge all C&i;

%end;

%end;

*----------------------------------------------------*

*Variance of the mean of arrays (*V*_0_*L*_1_)*

*----------------------------------------------------*

* The next set of statements compute the variance of the mean of arrays*

*---------------------------------------------------*;

meanX=sumX/&Pro1;

v0l1=(ssq(meanX) - ((sum(meanX)##2)/&Pro1))/(&Pro1-**1**);

*----------------------------------------------------*

*Covariance between parents and their offspring (*W*_r_) *

*----------------------------------------------------*

*The next set of statements compute the covariance between parents and their offspring*

*---------------------------------------------------*;

Wr=j(&Pro1,**1,0**);

diag=diag[,+];

do i=**1** to &Pro1;

Pi=X[,i];

W=Pi # diag;

Wr[i]=(sum(W) - (sumX[i] # sumDIAG/&Pro1))/(&Pro1-**1**);

end;

diff = Wr - Vr;

sum = Wr + Vr;

*----------------------------------------------------*

*Testing the significant of *b* from zero (0) – HO: *b* = 0*

*Testing the significance of *b* from unity (1) – HO: *b* =1*

*----------------------------------------------------*

*The next set of statements are used to test significance of slope from 0 and 1*

*---------------------------------------------------*;

b0=b/se;

b1=(**1**-b)/se;

free VWr VVr CVrWr;

*----------------------------------------------------*

*W_r_ – *V*_r_ graph*

*----------------------------------------------------*

*The next set of statements are for graphical analysis of the diallel using covariance between families and variance among families*

*---------------------------------------------------*;

Wri=(Vr # v0l0)##(**1**/**2**);

MeanWr=sum(Wr)/&Pro1;

MeanVr=sum(Vr)/&Pro1;

Wrei=MeanWr-(B#MeanVr)+(B#Vr);

Wreiprime=MeanWr-(**1**#MeanVr)+(**1**#Vr);

vfcp = j(&Pro1,**1**,**1**);

Wriqrt=quartile(Wri);

Vrqrt=quartile(Vr);

Intercept=MeanWr-(B#MeanVr);

free MeanWr MeanVr;

Graph=Wr ?/?/ Vr ?/?/ Wri ?/?/Wrei?/?/Wreiprime;

qrt=Wriqrt?/?/Vrqrt;

*----------------------------------------------------*

*Estimation of genetic components of variation*

*----------------------------------------------------*

*The next set of statements are for estimating *D*, *F*, *H*_1_, *H*_2_, and E*

*---------------------------------------------------*;

E=ems;

D=v0l0-E;

if D < **0** then D=**0**;

F=(**2**#v0l0)-(**4**#w0l01)-(**2**#(&Pro1-**2**)#E/&Pro1);

if F < **0** then F=**0**;

H1=v0l0-(**4**#w0l01)+(**4**#v1l1)-((**3**#&Pro1-**2**)#E/&Pro1);

if H1 < **0** then H1=**0**;

H2=(**4**#v1l1)-(**4**#v0l1)-(**2**#E);

if H2 < **0** then H2=**0**;

SH2=(**4**#DPP)-(**4**#(&Pro1-**1**)#E/(&Pro1##**2**));

if SH2 < **0** then SH2=**0**;

S2=(**1/2**)#(**1**/(&Pro1-**1**))#((ssq(Wr-Vr))-((sum(Wr-Vr)##**2**)/&Pro1));

if S2 < **0** then S2=**0**;

N1=&Pro1;

N2=&Pro1##**2**;

N3=&Pro1##**3**;

N4=&Pro1##**4**;

N5=&Pro1##**5**;

**%mend** first;

%***first***;

**RUN**;

## Examples of Hayman’s Diallel Analysis using SASHAYDIALL

To demonstrate usage and show key output from SASHAYDIALL, we reanalyzed data from a cabbage diallel experiment with reciprocals (Tanaka and Niikura, 2006) and also analyzed data from a multilocation maize diallel without reciprocals.

### Example 1: Cabbage Diallel

Details of the seven-by-seven cabbage diallel are found in Tanaka and Niikura (2006). We reanalyzed data of two parameters (width of the 15th wrapper leaf [W15] and leaf shape index of the 15th wrapper leaf [LSI15]). In the analysis of cabbage data, SASHAYDIALL performs the general ANOVA (Supplemental Fig. S1), which is the first requirement to test for significance among genotypes before proceeding with further analysis. For the cabbage diallel study, there were highly significant differences (P < 0.0001) among the genotypes, and based on this result, the user can proceed with interpretation of results from other analyses proposed by Hayman (1954b). For this data set, the SASHAYDIALL program detects the presence of reciprocals, and hence it computes ANOVA with items *a*, *b* (and its components), *c*, and *d*. The components *a*, *b*, *c*, and *d* are tested for significance using both their respective interaction with replication and the residual as the error term in the ANOVA. The user has to decide which output to use for interpretation, although Hayman (1954b) and Mather and Jinks (1971) recommended testing the significance of components *a* and *b* using their respective interaction with block as the error term. The ANOVA output for two traits, W15 and LSI15, (Supplemental Fig. S1) generated by SASHAYDIALL is similar to that presented in Table 3 of Tanaka and Niikura (2006), except for minor differences in estimation of some parameters for LSI15. The genetic parameters generated by SASHAYDIALL for the two traits in cabbage (Table 1) are nearly identical to those given in Table 4 of Tanaka and Niikura (2006). The genetic components of variation (*D*, *H*_1_, *H*_2_, *F*, and *h*_2_) and their SEs are computed by SASHAYDIALL to allow for a test of significance. The *W*_r_ –*V*_r_ graphs plotted by SASHAYDIALL (Supplemental Fig. S2) show the distribution of dominant and recessive genes among the parents, and these graphs are similar to those presented by Tanaka and Niikura (2006).

**Table 1:** Genetic parameters of two developmental characteristics (width of the 15th wrapper leaf [W15] and leaf shape index of the 15th wrapper leaf [LSI15]) in cabbage published in Tanaka and Niikura (2006)†, and estimates and their standard errors (± SE) using SASHAYDIALL.

Parameter‡	Published		SASHAYDIALL	
	W15	LSI15	W15	LSI15
*D*	–§	–	6.697 ± 3.399¶	0.010 ± 0.001
*H*_1_	–	–	49.812 ± 8.184	0.003 ± 0.001
*H*_2_	–	–	44.559 ± 7.212	0.002 ± 0.001
*E*	–	–	0.000 ± 8.156	0.003 ± 0.001
*F*	–	–	3.688 ± 1.202	0.002 ± 0.000
Average degree of dominance	2.74	0.51	2.73	0.54
Correlation between *W*_r_ + *V*_r_ and *Y*_r_	−0.90**	−0.73	−0.89**	−0.74
Broad-sense heritability (*H*^2^)	0.83	0.70	–0.82	0.71
Narrow-sense heritability (*h*^2^)	0.31	0.63	0.29	0.63

** Significant at the 0.01 probability level.

† Adapted with permission from the publisher of Breeding Science.

‡ *D*, component of variation due to additive effect of genes; *H*_1_, component of variation due to dominance effects of genes; *H*_2_, dominance component indicating asymmetry of positive and negative effects of genes; *F*, relative frequency of dominant and recessive alleles in the parents; *E*, environmental variation; *W*_r_, covariance between families within the *i*th array and their nonrecurrent parent; *V*_r_, the variance among family (F_1_ + reciprocal) means within an array; *Y*_r_, mean parental value.

§ Estimates not provided in Tanaka and Niikura (2006).

¶ Parameter estimates are presented with more accuracy for purposes of illustration only.

### Example 2: Maize Diallel Data from Multiple Locations

Hybrids without their reciprocals from a 13-by-13 maize diallel cross together with their parental inbred lines were evaluated at three locations in Kenya. The hybrid trial with 78 diallel hybrids and two check hybrids was laid out as an 8-by-10 α (0,1) lattice, whereas the parental trial with 13 parents and two check inbred lines was laid out as a three-by-five α (0,1) lattice with two replications. Days to anthesis (DTA, days from planting to when 50% of the plants had shed pollen) were recorded for the hybrids and inbred lines and the data analyzed using SASHAYDIALL. Results of the general and genetic effects ANOVA by location and across locations are presented in Table 2. There were significant differences among genotypes, and therefore further analysis according to Hayman (1954b) is valid. Without reciprocals, SASHAYDIALL only computes components a and b. In this example both *a* and *b* gene effects were highly significant (*P* < 0.001) for DTA at each location. Significance of component b indicates presence of dominance for this trait.

**Table 2:** Analysis of variance of a 13-by-13 diallel table for days to anthesis of maize evaluated at three locations in Kenya using SASHAYDIALL.

Item	df	Kiboko, 2009		Kiboko, 2012		Embu, 2012		Kakamega, 2012		Across locations	
		MS†	F-test	MS	F-test	MS	F-test	MS	F-test	MS	F-test
*a‡*	12	6.27	14.98***	14.25	26.41***	32.08	12.04***	18.91	13.42***	40.79	2.28*
*b_1_*	1	20,945.04	12,948.19***	21,933.59	30,508.4***	25,095.64	5,754.31***	26,579.56	18,861.17***	68,868.40	2.99NS§
*b_2_*	12	4.67	9.02***	11.41	15.73***	17.16	4.60***	16.59	11.77***	11.37	1.69NS
*b_3_*	65	5.74	14.16***	7.10	12.12***	9.45	4.25***	9.12	6.47***	0.81	1.11NS
*b*	78	274.03	625.68***	288.87	474.24***	332.26	133.90***	350.92	249.01***	885.35	2.***
*a* × Environment (Env)	36									17.92	0.58NS
*b*_1_ × Env	3									23,042.24	742.32***
*b*_2_, × Env	36									6.74	0.22NS
*b*_3_ × Env	195									0.73	0.02NS
*b* × Env	234									297.06	9.57**
Genotype (Gen)	90	10.66	13.98***	15.56	14.29***	25.02	5.68***	20.30	7.92***	59.43	26.96***
Environment	3									3,460.39	13.32***
Gen x Env	270									4.03	1.83***
Replication(Env)	4									4.74	2.15NS
Error	360									31.04	

*,**,*** Significant at the 0.05, 0.01, and 0.001 probability levels, respectively.

† MS, mean square.

‡ *a*, additive effect; b, dominance effect; *b*_1_, measure of directional dominance; *b*_2_, measure of ambidirectional dominance; *b*_3_, residual dominance.

§ NS, not significant.

**Table 3:** Genetic component estimates for days to male flowering in a 13-by-13 half-diallel of maize across three locations estimated using SASHAYDIALL

Parameter†	Estimate ± SE
*D*	5.717 ± 0.629‡
*H*_1_	11.690 ± 1.225
*H*_2_	11.069 ± 1.009
*h*_2_	125.420 ± 0.674
*F*	1.912 ± 1.415
*E*	1.099 ± 0.168
Mean degree of dominance	1.430
Proportion of dominance	0.230
Proportion of dominant and recessive genes in parents	1.265
Number of groups of genes which control character	11.331
Correlation (*r*) between *W*_r_ + *V*_r_ and *Y*_r_	0.935***
Prediction for measurement of completely dominant and recessive parents	0.874
Broad-sense heritability (*H*^2^)	0.819
Narrow-sense heritability (*h*^2^)	0.364

*** Significant at the 0.001 probability level.

† *D*, component of variation due to additive effect of genes; *H*_1_, component of variation due to dominance effects of genes; *H*_2_, dominance component indicating asymmetry of positive and negative effects of genes; *h*_2_, overall mean dominance effect of heterozygous loci; *F*, relative frequency of dominant and recessive alleles in the parents; *E*, environmental variation; *W*_r_, covariance between families within the *i*th array and their nonrecurrent parent; *V*_r_, the variance among family (F_1_ + reciprocal) means within an array; *Y*_r_, mean parental value.

‡ Parameter estimates are presented with more accuracy for purposes of illustration only.

**Fig. 2:**
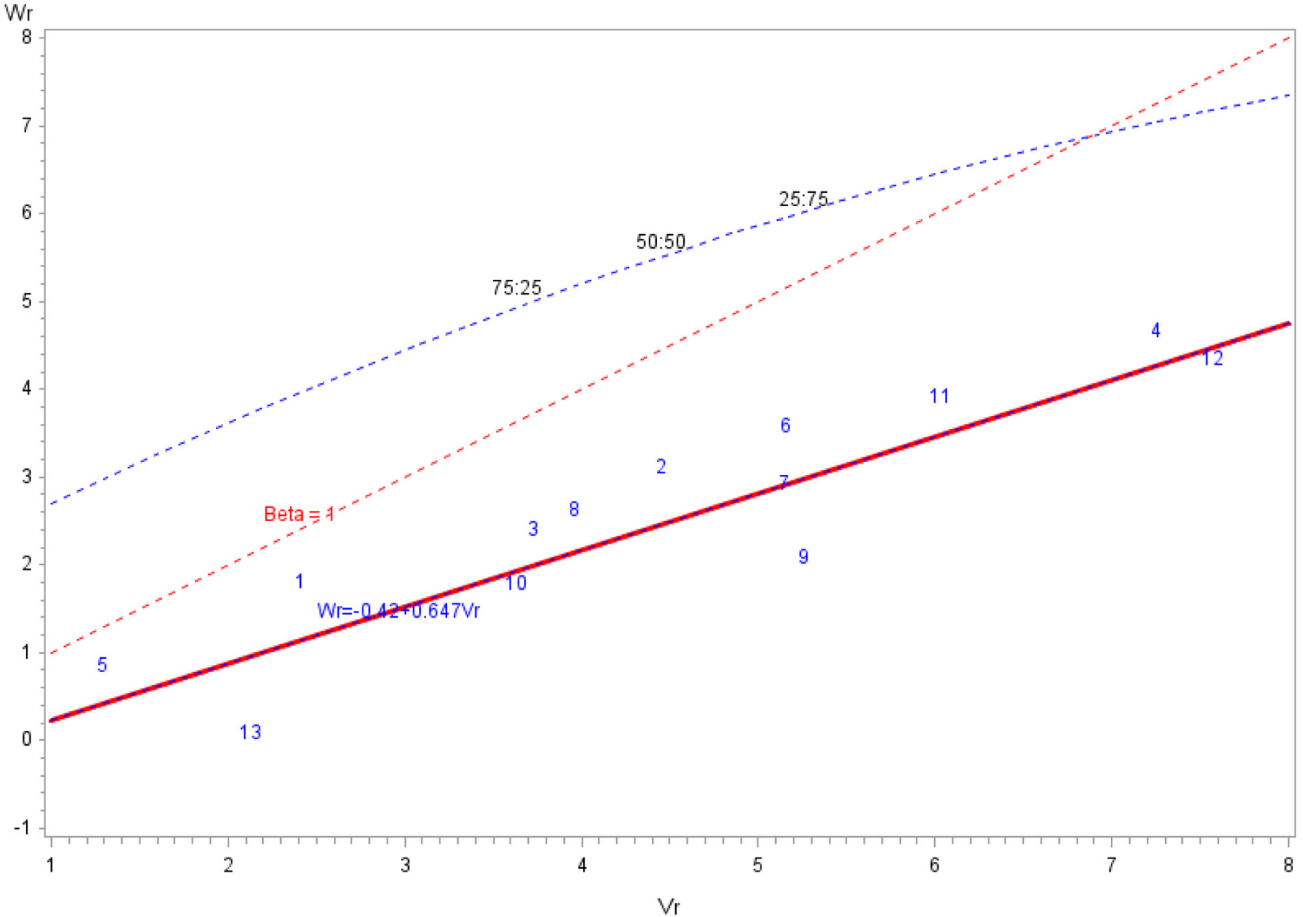
Hayman’s *W*_r_ − *V*_r_ graph for days to anthesis in a 13-by-13 maize diallel across three locations in Kenya plotted using SASHAYDIALL. *V*_r_ is the variance among family (F_1_ + reciprocal) means within an array, and *W*_r_ is the covariance between families within the *i*th array and their nonrecurrent parent

The genetic parameters for DTA are estimated by SASHAYDIALL across locations (Table 3) and for individual locations (Supplemental Table S_2_). In this example, genetic components *D*, *H*_1_, *H*_2_, and *h*_2_ were all significant, and dominance genetic variance was larger than additive genetic variance across locations. Significance of both *D* and *H* components suggests that DTA is controlled by both additive and dominant effects. Furthermore, an estimate of the number of groups of genes that control DTA and exhibit dominance and heritability (broad and narrow sense) are provided among others (Table 3, Supplemental Table S2). The relationship between covariance of parental inbred lines and hybrids (*W*_r_) and variance of the F_1_ hybrids is shown in the *W*_r_ − *V*_r_ graph, which gives the ranking of inbred lines for frequency of dominant alleles for DTA across locations (Fig. 2) and individual locations (Supplemental Fig. S3a and S3b). In addition, the *W*_r_ − *V*_r_ graph plotted by SASHAYDIALL shows the proportions of dominant to recessive genes (75:25, 50:50, and 25:75%) (Fig. 2, Supplemental Fig. S3a and S3b). The SASHAYDIALL program also plots a graph of *W*_r_ + *V*_r_ against *Y*_r_ (Supplemental Fig. S4).

## DISCUSSION

Diallel mating designs provide important genetic information useful in a breeding program. The SASHAYDIALL program described in this paper is based on models for analysis of a diallel developed by Jinks and Hayman (1953) and Hayman (1954a, 1954b), and extended by Morley Jones (1965) for the half-diallel, which is frequently used. This method of analysis has been used to analyze diallel cross data sets with and without reciprocals in many crops. Several genetic components estimated by Hayman’s method are related to the components in the method proposed by Griffing (1956), and hence output from the two methods of analysis can be compared by the breeder.

The SASHAYDIALL program is user friendly, as the user only needs to specify the location and name of the file with data to be analyzed, provide the variables required for the analysis, and indicate whether to analyze data from single or multiple locations. Breeders typically evaluate progenies from diallel crosses in multiple locations, and SASHAYDIALL can handle analysis of such data easily. The analytical procedure in SASHAYDIALL is automated, as the program automatically detects the number of parents in the diallel cross data set and presence or absence of reciprocals and computes the number of genotypes. The significance of genetic components *a*, *b*, *c*, and *d* is tested using both the pooled error and block interaction as the error terms (Mather and Jinks, 1971). The user should decide which output to use for interpretation, although Hayman (1954b) and Mather and Jinks (1971) provided recommendations that can be followed by the user. The genetic (*D*, *H*_1_, *H*_2_, *F*, and *h*_2_) and environmental (*E*) components are computed for single and multiple locations together with their SEs, which enables a test for their significance.

Estimates of *h*_2_ and *H*_2_, mean degree of dominance, and the number of groups of genes that control the trait of interest are computed to aid in the interpretation of the inheritance of a trait by the breeder. The *W*_r_ − *V*_r_ graph that provides an insight into the order of dominance and an estimate of the proportions of dominant to recessive alleles among the parents is generated. The *W*_r_ and *V*_r_ output can be used in other software to generate the *W*_r_ − *V*_r_ graph with the limiting parabola, which is not provided for in this program. The plot of *W*_r_ + *V*_r_ against *Y*_r_ generated by SASHAYDIALL can give an indication of the effect of dominant or recessive alleles on expression of a trait.

In summary, the SASHAYDIALL program was written to provide user-friendly, freely available analytical software for Hayman’s diallel analysis. SASHAYDIALL has an advantage that the user does not have to indicate the number of parents, as this is automatically detected and the program can analyze data from multiple environments. The SASHAYDIALL program generates comprehensive output that is easy to understand for proper genetic interpretation of the inheritance of a trait by the breeder. This program should revive the nterest in application of Hayman’s (1954b) method of diallel analysis because of ease of use. The SASHAYDIALL program runs in SAS (SAS Institute, 2014), which is among the most powerful and widely used software for statistical analysis. An interested user does not need any knowledge of the SAS/IML language to analyze data with this program. The SASHAYDIALL program is not computationally intensive and should therefore run on slower computers. Users are advised against making any changes to the program code.

## AVAILABILITY

The SASHAYDIALL program described in this paper was developed and tested in SAS version 9.4 (SAS Institute, 2014), but it should work with SAS version 9.0 and upward. The SASHAYDIALL program is freely available to interested users from the corresponding author or from the CIMMYT Biometrics and Statistics Unit software repository (http://hdl.handle.net/11529/10548045). The data set used for the maize diallel example and corresponding output will be provided on request.

## Abbreviations

*a*, additive genetic effects; *b*, dominant genetic effects; *c*, average maternal effects of each parental line; *d*, variation in the reciprocal differences not attributed to *c*; *D*, component of variation due to additive effect of genes; DTA, days to anthesis; *F*, relative frequency of dominant and recessive alleles in the parents; GCA, general combining ability; *H*_1_, component of variation due to dominance effects of genes; *H*_2_, dominance component indicating asymmetry of positive and negative effects of genes; *h*_2_, overall mean dominance effect of heterozygous loci; *H*^2^, broad-sense heritability; *h*_2_, narrow-sense heritability; LSI15, leaf shape index of the 15th wrapper leaf; SCA, specific combining ability; *V*_r_, the variance among family (F_1_ + reciprocal) means within an array; *V*_ri_, expected variance; *W*_r_, covariance between families within the ith array and their nonrecurrent parent; *W*_ri_, parent–offspring covariance; W15, width of the 15th wrapper leaf; *Y*_r_, mean parental value.
