# Supplementary material for: SASHAYDIALL: A SAS Program for Hayman’s Diallel Analysis
Source: Crop Sci. 2018 Jun 14;58(4):1605–15. doi: 10.2135/cropsci2018.01.0047 (PMC7680942; doi:10.2135/cropsci2018.01.0047)
Supplement: Supplementary file 1 [file CROPSCI-58-04-1605-s001.pdf]

Type 3 Analysis of Variance								
**Source**	**DF**	**Sum of Squares**	**Mean Square**	**Expected Mean Square**	**Error Term**	**Error DF**	**F Value**	**Pr > F**
**Genotype**	48	1847.847041	38.496813	Var(Residual) + Q(Genotype)	MS(Residual)	48	5.13	<.0001
**Replication**	1	1.361250	1.361250	Var(Residual) + 49 Var(Replication)	MS(Residual)	48	0.18	0.6720
**Residual**	48	360.035000	7.500729	Var(Residual)	.	.	.	.

ANALYSIS OF VARIANCE BY ENVIRONMENT, OF THE DIALLEL (HAYMAN'S METHOD) USING RESIDUAL AS ERROR TERM

ENVIRONMENT = 1

Source	DF	SS	MS	FValue	Probability_F
a	6	603.0934	100.5156	13.4008	0.0000
b1	1	788.7092	788.7092	105.1510	0.0000
b2	6	132.4920	22.0820	2.9440	0.0158
b3	14	169.4300	12.1021	1.6135	0.1097
b	21	1090.6312	51.9348	6.9240	0.0000
c	6	33.7829	5.6305	0.7507	0.6119
d	15	120.3396	8.0226	1.0696	0.4076

A

Type 3 Analysis of Variance								
**Source**	**DF**	**Sum of Squares**	**Mean Square**	**Expected Mean Square**	**Error Term**	**Error DF**	**F Value**	**Pr > F**
**Genotype**	48	0.566835	0.011809	Var(Residual) + Q(Genotype)	MS(Residual)	48	2.99	0.0001
**Replication**	1	0.002351	0.002351	Var(Residual) + 49 Var(Replication)	MS(Residual)	48	0.59	0.4444
**Residual**	48	0.189749	0.003953	Var(Residual)	.	.	.	.

ANALYSIS OF VARIANCE BY ENVIRONMENT, OF THE DIALLEL (HAYMAN'S METHOD) USING RESIDUAL AS ERROR TERM

ENVIRONMENT = 1

Source	DF	SS	MS	FValue	Probability_F
a	6	0.3819	0.0637	16.1030	0.0000
b1	1	0.0153	0.0153	3.8719	0.0549
b2	6	0.0393	0.0065	1.6564	0.1524
b3	14	0.0710	0.0051	1.2820	0.2532
b	21	0.1255	0.0060	1.5123	0.1181
c	6	0.0065	0.0011	0.2734	0.9467
d	15	0.0529	0.0035	0.8915	0.5776

B

Supplemental Fig. S1. ANOVA output from SASHAYDIALL for characters W15 (A) and LSI15 (B) in cabbage (data provided by Dr. Tanaka for paper published in Breeding Science 56:147–153, 2006).

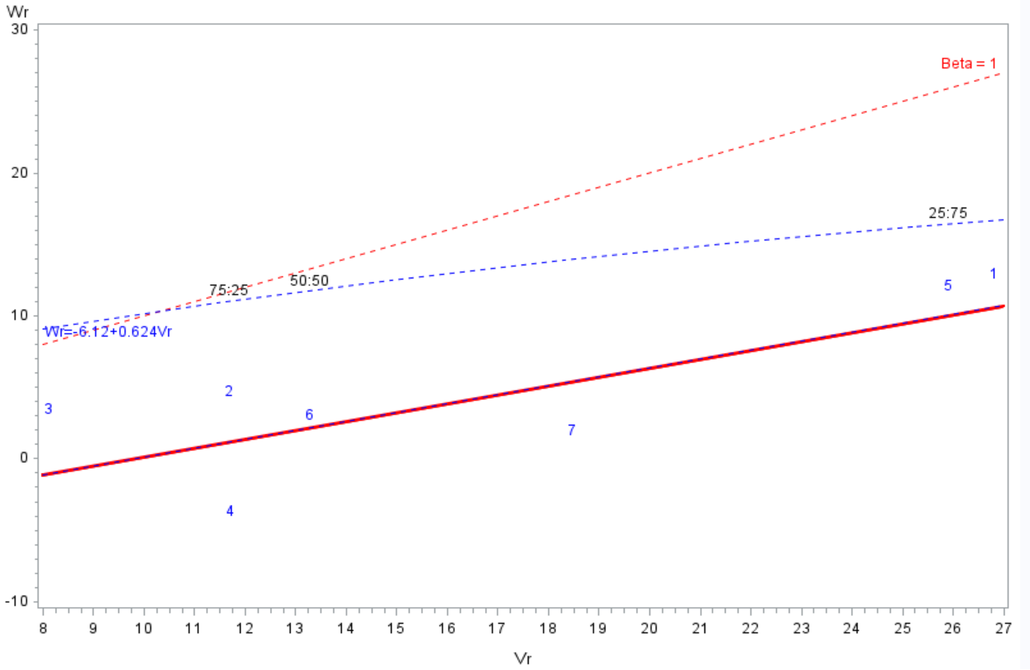

A

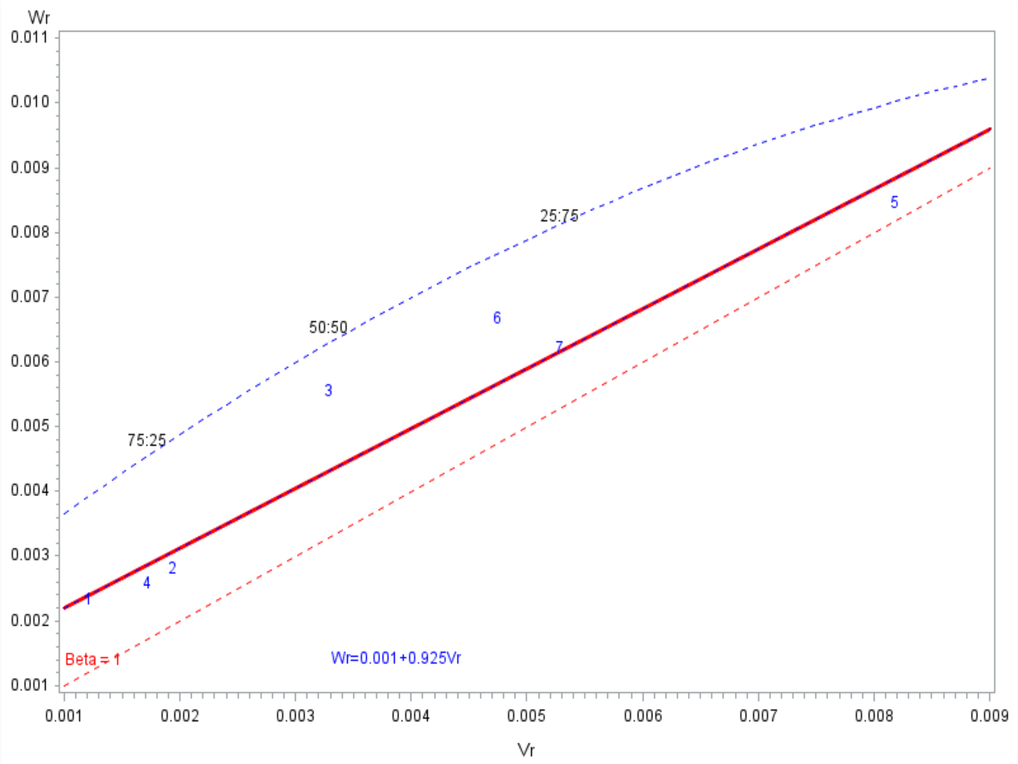

B

Supplemental Fig. S2. Hayman's W_r–V_r graph for characters W15 (A) and LSI15 (B) in cabbage plotted using SASHAYDIALL (data provided by Dr. Tanaka for paper published in Breeding Science 56:147–153, 2006).

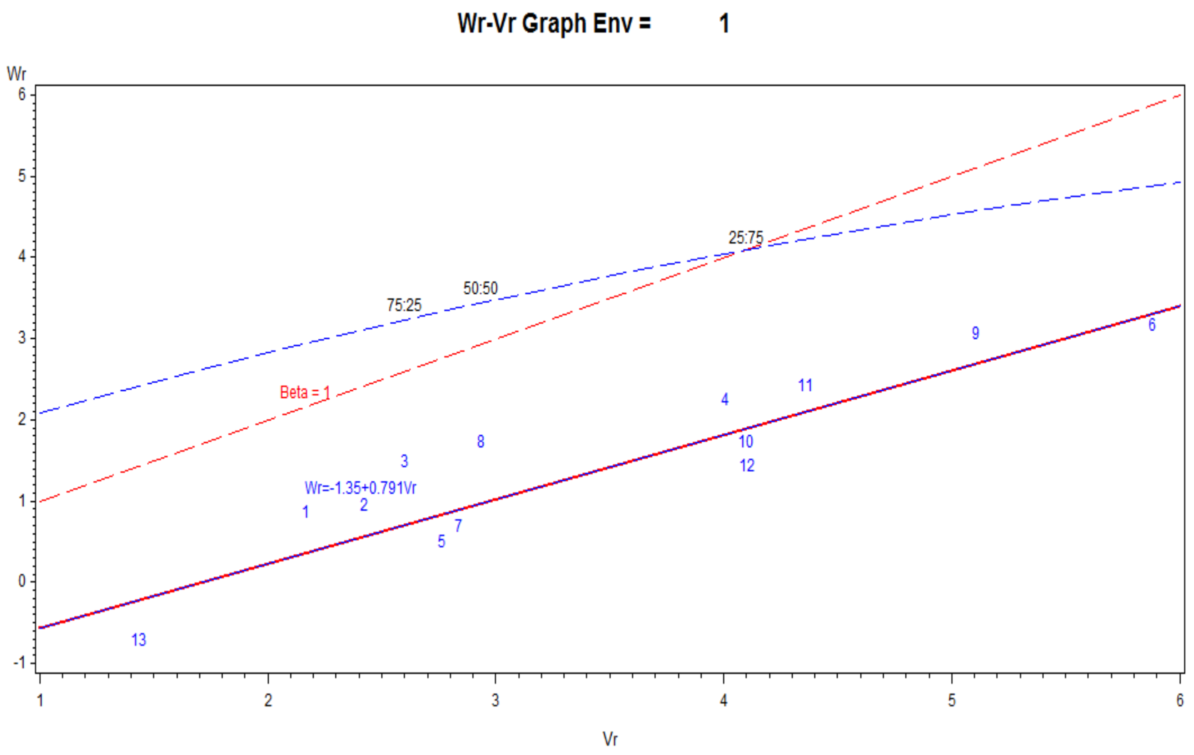

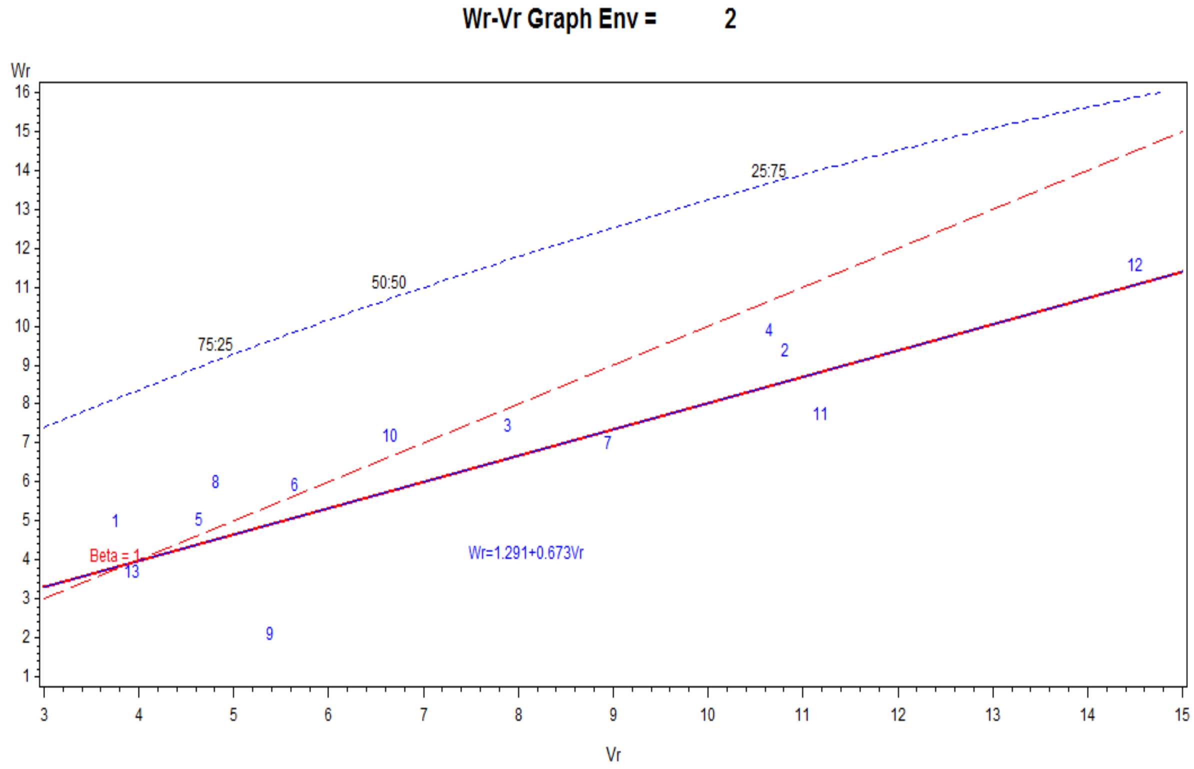

Supplemental Fig. 3a. Hayman's W_r–V_r graph for DTA in a 13 × 13 maize diallel at two locations in Kenya (Env 1=Kiboko 2009; Env 2=Embu 2012) plotted using SASHAYDIALL.

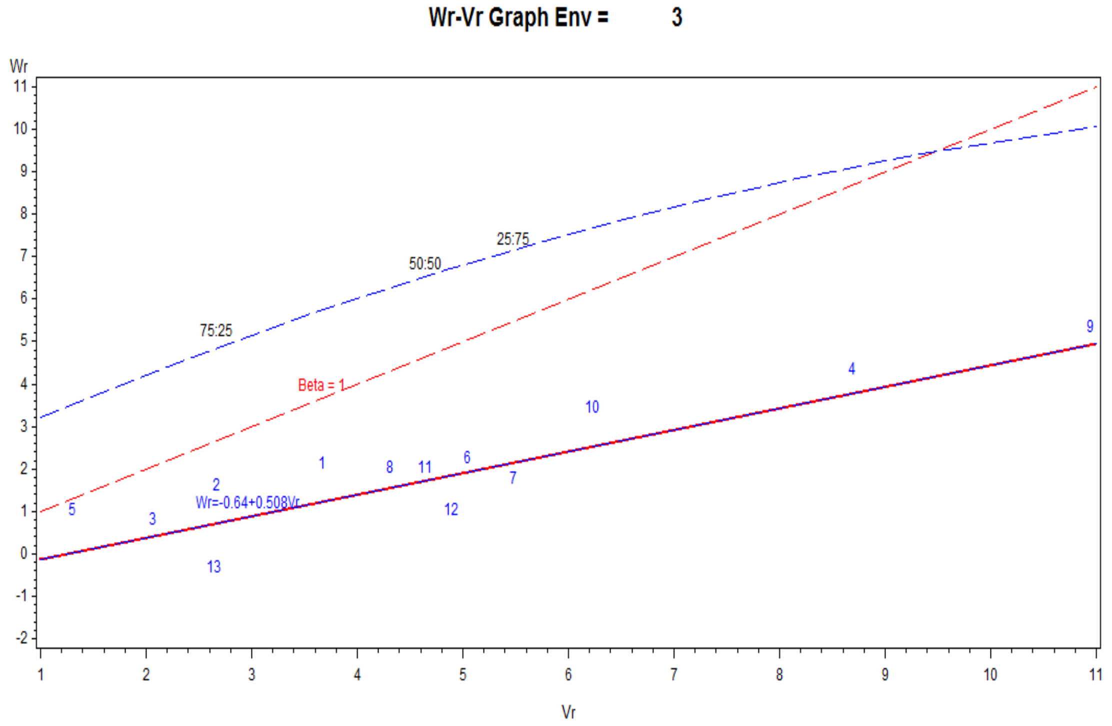

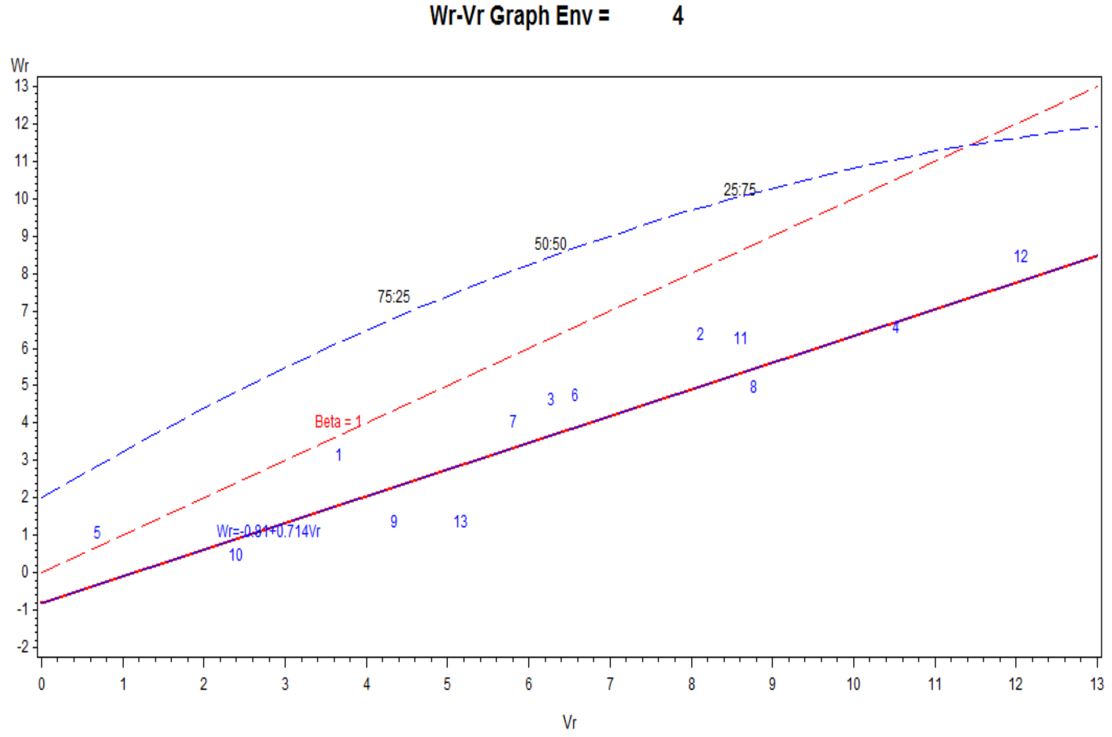

Supplemental Fig. 3b. Hayman's W_r–V_r graph for DTA in a 13 × 13 maize diallel at two locations in Kenya (Env 3=Kiboko 2012; Env 4=Kakamega 2012) plotted using SASHAYDIALL.

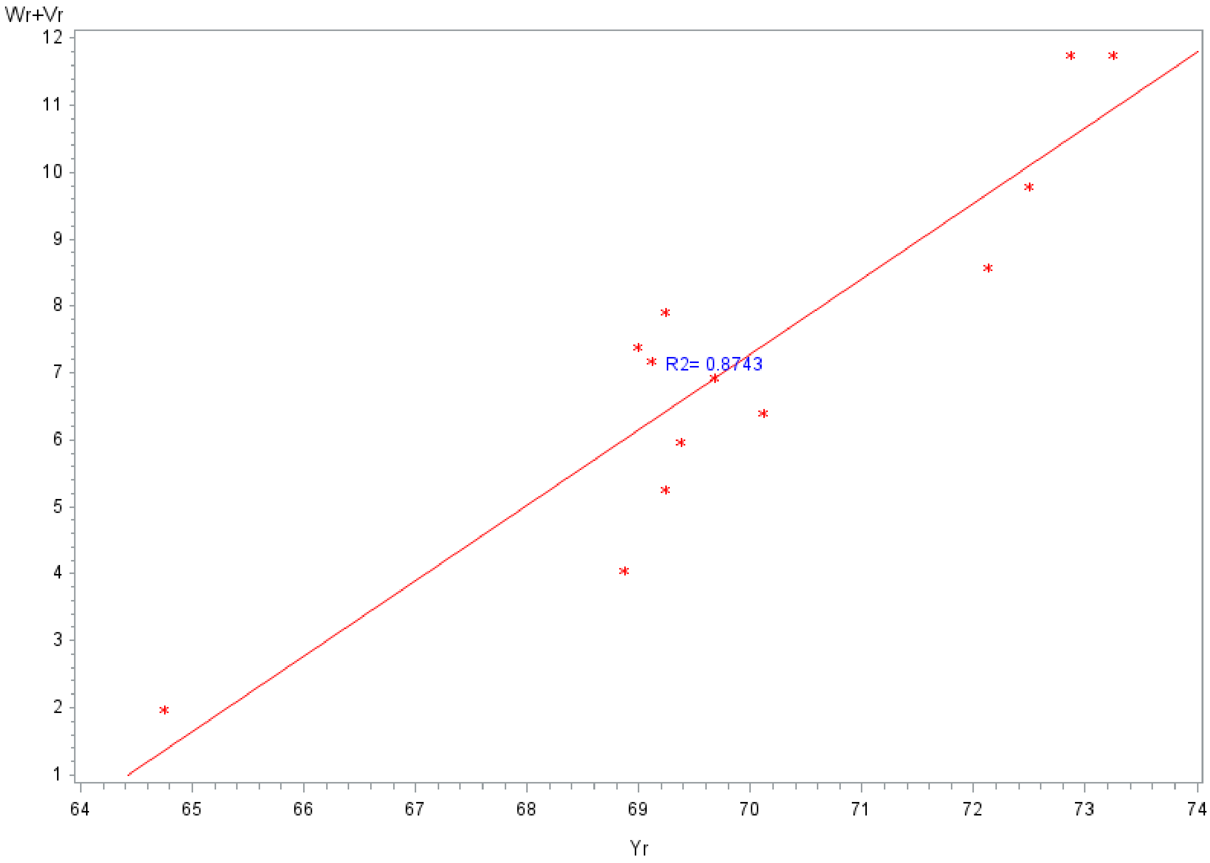

Supplemental Fig. S4. A $W_r + V_r$ vs Y_r graph for DTA in a 13 × 13 maize diallel across three locations in Kenya plotted using SASHAYDIALL.

Supplemental Table S1. Sample data arrangement of a multi-environment F_1 diallel hybrid trial for analysis using SASHAYDIALL.

P1	P2	REP	ENTRY	ENV	AD
1	2	1	1	1	71
1	3	1	2	1	73
1	4	1	3	1	70
1	5	1	4	1	68
1	6	1	5	1	71
1	7	1	6	1	71
1	8	1	7	1	71
1	9	1	8	1	70
1	10	1	9	1	72
1	11	1	10	1	71
1	12	1	11	1	73
1	13	1	12	1	72
2	3	1	13	1	72
………………. more data …………….					
4	5	2	34	2	67
4	6	2	35	2	68
4	7	2	36	2	68
4	8	2	37	2	68
4	9	2	38	2	66
4	10	2	39	2	66
4	11	2	40	2	70
4	12	2	41	2	70
4	13	2	42	2	64
5	6	2	43	2	66
5	7	2	44	2	65
5	8	2	45	2	66

P1	P2	REP	ENTRY	ENV	AD
1	1	1	1	3	75
2	2	1	2	3	75
3	3	1	3	3	75
4	4	1	4	3	78
5	5	1	5	3	75
6	6	1	6	3	82
7	7	1	7	3	75
8	8	1	8	3	75
9	9	1	9	3	75
10	10	1	10	3	82
11	11	1	11	3	85
12	12	1	12	3	71
13	13	1	13	3	71
………………. more data …………….					
1	1	1	1	4	73
2	2	1	2	4	74
3	3	1	3	4	75
4	4	1	4	4	76
5	5	1	5	4	66
6	6	1	6	4	73
7	7	1	7	4	71
8	8	1	8	4	78
9	9	1	9	4	71
10	10	1	10	4	73
11	11	1	11	4	76
12	12	1	12	4	78

Supplemental Table S2. Genetic component estimates for days to male flowering in a 13-by-13 half-diallel of maize at four locations estimated using SASHAYDIALL.

Parameter†	Kiboko, 2009	Kiboko, 2012	Embu, 2012	Kakamega, 2012
D	3.655 ± 0.362‡	8.654 ± 1.081	15.160 ± 1.206	10.015 ± 0.974
H1	11.217 ± 0.705	19.691 ± 2.105	15.813 ± 2.349	18.144 ± 1.897
H2	10.115 ± 0.580	15.179 ± 1.733	14.048 ± 1.934	16.800 ± 1.562
h2	86.852 ± 0.388	139.339 ± 1.158	129.786 ± 1.292	150.818 ± 1.044
F	1.959 ± 0.814	10.235 ± 2.431	5.380 ± 2.713	5.430 ± 2.192
E	0.393 ± 0.097	0.538 ± 0.289	2.231 ± 0.322	1.302 ± 0.260
Mean degree of dominance	1.752	1.508	1.021	1.346
Proportion of dominance	0.225	0.193	0.222	0.231
Proportion of dominant and recessive genes in parents	1.361	2.289	1.421	1.504
Number of groups of genes which control character	8.587	9.180	9.239	8.977
Correlation (r) between $W_r + V_r$ and Y_r	0.902***	0.841***	0.888***	0.904***
Prediction for measurement of completely dominant and recessive parents	0.814	0.708	0.789	0.817
Broad-sense heritability (H^2)	0.909	0.907	0.806	0.846
Narrow-sense heritability (h^2)	0.324	0.253	0.501	0.350

*** Significant at the 0.001 probability level.

†*D*, component of variation due to additive effect of genes; H_1, component of variation due to dominance effects of genes; H_2, dominance component indicating asymmetry of positive and negative effects of genes; h2, overall mean dominance effect of heterozygous loci; F, relative frequency of dominant and recessive alleles in the parents; E, environmental variation; W_r, covariance between families within the *i*th array and their nonrecurrent parent; V_r, the variance among family (F_1 + reciprocal) means within an array; Y_r, mean parental value.

‡ Parameter estimates are presented with more accuracy for purposes of illustration only.
